# Dataset on determinants of intention and investment behaviour amongst young Indonesian millennials

**DOI:** 10.1016/j.dib.2020.106083

**Published:** 2020-07-25

**Authors:** Ummu Salma Al Azizah, Herri Mulyono

**Affiliations:** University of Muhammadiyah Prof. DR. HAMKA, Jakarta, Indonesia

**Keywords:** Financial literacy, Self-control, Peer-influence, Intention, Investment behaviour, Young millennials

## Abstract

The dataset examines three factors (i.e. financial literacy, self-control, peer-influence) that influence investment behaviour amongst young Indonesian millennials. Using a non-probability sampling technique, a total of 265 young millennials attending Investment Gallery seminar and workshop events in Jakarta, Indonesia completed an investment behaviour survey. The reflective measurement was performed using the Partial Least Square Structural Equation Model (PLS-SEM) to the screened data from the survey (*N* = 213). The measurement includes the evaluation of outer loading, internal reliability, and convergent reliability. The final equation model was evaluated and estimated using SmartPLS v.3.3.2 program. The dataset is beneficial for companies seeking for potential investors from young millennials. The dataset also provides valuable insights for the Indonesian Ministry of Education and Culture (MoEC) and universities, particularly the faculty of economy and business in preparing their students with the financial literacy and investment knowledge.

Specification table

**Table:** 

**Subject**	Economy and Business
**Specific subject area**	Economy and Business
**Type of data**	Table
**How data were acquired**	A survey method was carried out to collect the quantitative data
**Data format**	Raw
	Analysed
	Filtered
**Parameters for data collection**	The data on young Indonesian millennials' financial literacy, self-control, peer-influence and investment behaviour were collected using an investment behaviour questionnaire.
**Description of data collection**	A total of 265 young Indonesian millennials attending Investment Gallery seminar and workshop events completed an investment behaviour survey. After the screening process, 213 data were evaluated using the Partial Least Square Structural Equation Model (PLS-SEM)
**Data source location**	Jakarta, Indonesia
**Data accessibility**	The data available in Mendeley Data: https://data.mendeley.com/datasets/d5kck749zs/2

## Value of the data

• The dataset describes financial literacy, self-control, peer-influence and investment behaviour amongst young Indonesian millennials who attended Investment Gallery seminar and workshop events in a private university in Jakarta, Indonesia. The dataset is useful for companies who are seeking potential investors from the young Indonesian millennials
• The dataset is also useful in providing insights for the Indonesian Ministry of Education and Culture (MoEC) and universities, particularly the faculty of economy and business to prepare their students with financial literacy and investment knowledge. More importantly, the university teachers and curriculum designers can use the information from the dataset to develop relevant course syllabus that promotes financial literacy and investment knowledge.
• In the field of behavioural economic research, the dataset helps researchers to address the current issues related to the roles of individual financial literacy and financial behaviour (e.g. individuals’ making a decision about consumption, saving, debts, investment) in maintaining financial stability in both micro and macro economy settings.
• The dataset might also be useful for researchers who are interested in evaluating the impact of young millennials' investment behaviour on the greater national economy in Indonesian settings.

## Data description

A survey method was employed to gather the primary data. A five-point Likert scale questionnaire with 30 items was adapted from literature [1,2] and was classified into four variables. The three exogenous variables are financial literacy or FL (6 items), self-control or SC (9 items), peer-influence or PI (6 items), and an endogenous variable of investment behaviour or IB (9 items). In addition to these variables, demographic information was gathered from the participant (see Table 1). The adapted questionnaire was translated into the native of Bahasa Indonesia to allow participants to comprehend the information. The questionnaire is provided as a supplementary file of this paper.

**Table 1:** Demographic information.

Demographic		N	Percentage
Gender	Male	68	31.92
	Female	145	68.08
Age	17–20 years old	142	66.67
	21–25 years old	69	32.39
	26–30 years old	2	0.94
Allowance	< 500 K IDR	125	58.69
	500 K - 1,000k IDR	70	32.86
	1000 K IDR <	18	8.45

*Note.* 1 *K* *=* *1000; $USD 1* *=* *14,740 IDR*.

## Experimental design, materials, and methods

The primary data collection was performed through an online survey. Using a non-probability sampling technique, a total of 265 young Indonesian millennials attending the Investment Gallery seminar and workshop events in a private university in Jakarta, Indonesia last 11 January 2020 completed an online version of the investment behaviour survey. Prior to the distribution, the participants were given a brief explanation about the survey and the instruction on how to complete it. The collected data then were tabulated using an excel application and were screened for missing values and outliers. The residual values above 1.96 were considered outliers [3]. Literature [3,4] has suggested that outliers can influence the parametric statistics and statistical significance result. Of 265 data, 52 data were found to be outliers and thus were removed. Garson [3] argues that the removal of outliers can improve robustness of PLS-SEM results. The remaining 213 data were analysed and characterised as in Table 1 below.

The normality assessment was performed by evaluating Skewness and Kurtosis [5]. As shown in Table 2, all the data fit the acceptable range of the Skewness and Kurtosis values. The Skewness and Kurtosis values were observed to be normal (the Skewness values ranged between −1 and 1, the Kurtosis values were between −2 and 2), indicating the data were normally distributed.

**Table 2:** Mean, Standard Deviation, Skewness, and Kurtosis.

Items	Mean	Standard Deviation	Kurtosis	Skewness
FL1	3.117	0.799	0.702	−0.438
FL2	3.610	1.123	−0.117	−0.709
FL3	3.310	1.011	−0.339	−0.296
FL4	3.376	0.993	−0.039	−0.492
FL5	3.089	0.809	0.063	0.050
FL6	3.404	0.991	−0.133	−0.357
PI1	3.362	0.902	0.261	−0.199
PI2	3.056	0.870	−0.119	0.062
PI3	3.000	0.856	0.506	−0.091
PI4	3.329	0.922	−0.280	0.024
PI5	2.915	0.936	−0.108	0.204
PI6	3.150	0.907	0.385	−0.150
SC1	2.408	1.205	−0.466	0.597
SC2	2.244	1.161	−0.435	0.671
SC3	2.113	1.302	−0.154	1.022
SC4	2.615	1.253	−0.793	0.371
SC5	2.563	1.101	−0.369	0.549
SC6	2.479	1.232	−0.667	0.542
SC7	2.690	1.047	−0.427	0.228
SC8	2.676	1.072	−0.580	0.146
SC9	3.047	1.091	−0.534	0.059
IB1	3.516	1.086	−0.431	−0.43
IB2	3.812	1.223	0.054	−0.97
IB3	3.836	1.269	−0.031	−1.005
IB4	3.653	1.118	0.016	−0.763
IB5	3.502	1.095	−0.502	−0.341
IB6	3.690	1.006	0.617	−0.907
IB7	3.620	1.075	0.239	−0.747
IB8	3.596	1.103	−0.226	−0.563
IB9	2.624	1.083	−0.163	0.546

**Fig 1:**
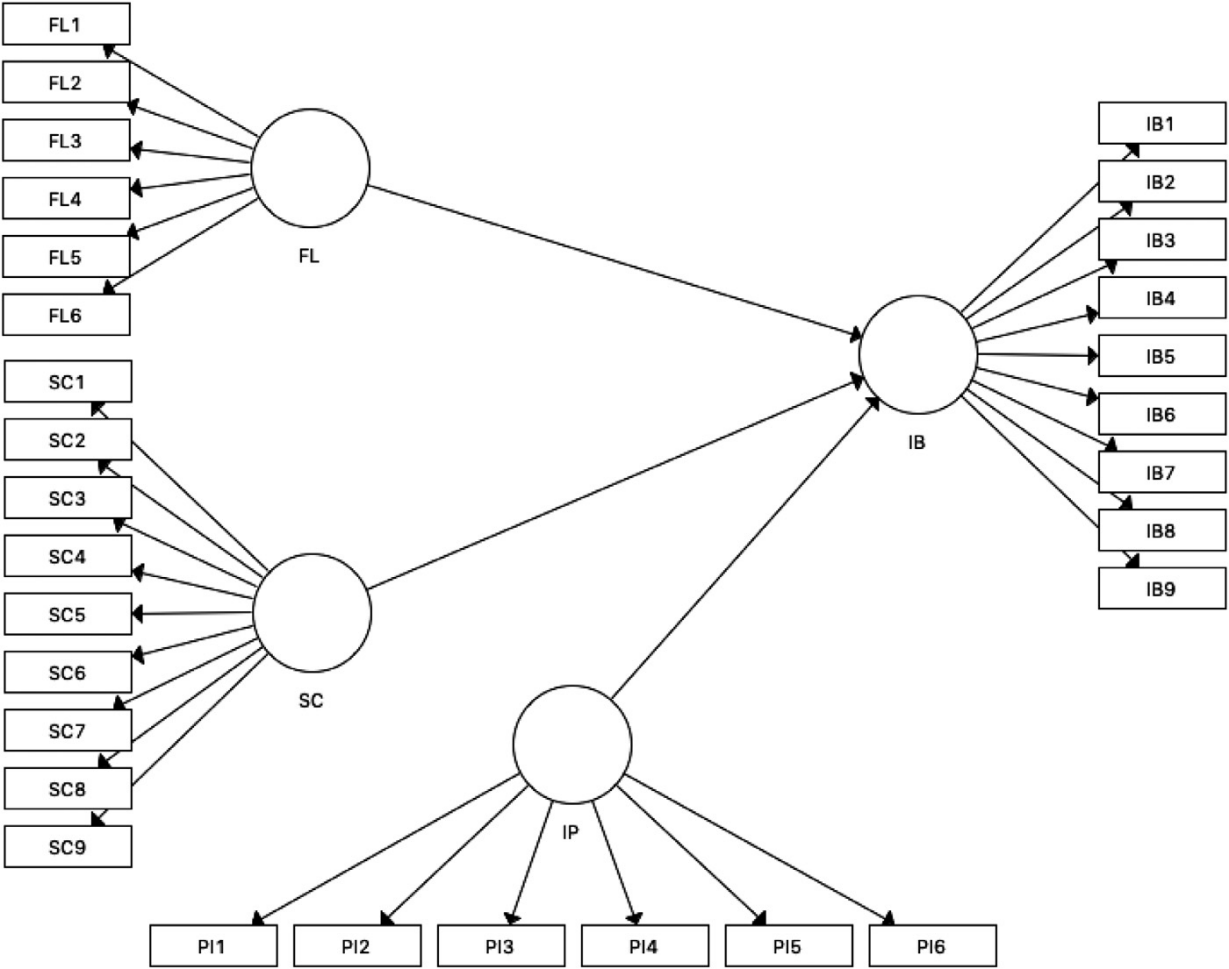
Proposed Model.

The reflective measurement of the remaining 213 data was performed using the Partial Least Square Structural Equation Model (PLS-SEM) with SmartPLS [6]. The proposed model (Fig. 1) was measured in line with the reflective measurement criteria [4]. First, the composite reliability (CR) and Cronbach's alpha are expected to be higher than 0.70, and the outer loading should be above 0.70. Third, the accepted value of the convergent reliability should outweigh 0.50. Table 3 presents the result of PLS-SEM algorithm analysis.

**Table 3:** The result of PLS-SEM algorithm analysis.

Variable	Item	Loading	Cronbach's Alpha	CR	AVE
Financial literacy (FL)	FL1	0.578	0.83	0.874	0.552
	FL2	0.884			
	FL3	0.729			
	FL4	0.869			
	FL5	0.384			
	FL6	0.872			
Peer-influence (PI)	PI1	0.862	0.538	0.556	0.249
	PI2	0.253			
	PI3	0.315			
	PI4	0.24			
	PI5	−0.025			
	PI6	0.728			
Self-control (SC)	SC1	0.737	0.897	0.915	0.553
	SC2	0.847			
	SC3	0.843			
	SC4	0.728			
	SC5	0.822			
	SC6	0.809			
	SC7	0.617			
	SC8	0.775			
	SC9	0.405			
Investment behaviour (IB)	IB1	0.779	0.892	0.874	0.552
	IB2	0.867			
	IB3	0.893			
	IB4	0.872			
	IB5	0.697			
	IB6	0.886			
	IB7	0.893			
	IB8	0.815			
	IB9	−0.236			

The result of PLS-SEM algorithm analysis showed that three variables (i.e. FL, SC and IB) met the reflective measurement criteria. The Cronbach's alpha and composite reliability (CR) values of these variables were reported higher than 0.70. The convergent reliability, which was examined through the average variance extracted (AVE), was observed higher than 0.50. However, the other exogenous variable of Peer-influence (PI) failed to meet the criteria and thus was removed from further analysis. Furthermore, Hair, Hult, Ringle, and Sarstedt [4] argue that the item loadings between 0.40 and 0.70 should be considered for deletion if only the deletion can increase the composite reliability. Five items (i.e. FL1, FL5, SC7, SC9 and IB9) were removed due to insufficient outer loading values. Second PLS-SEM algorithm analysis was performed after the item deletion, and the results revealed an increase in CR and AVE value as in Table 4.

**Fig 2:**
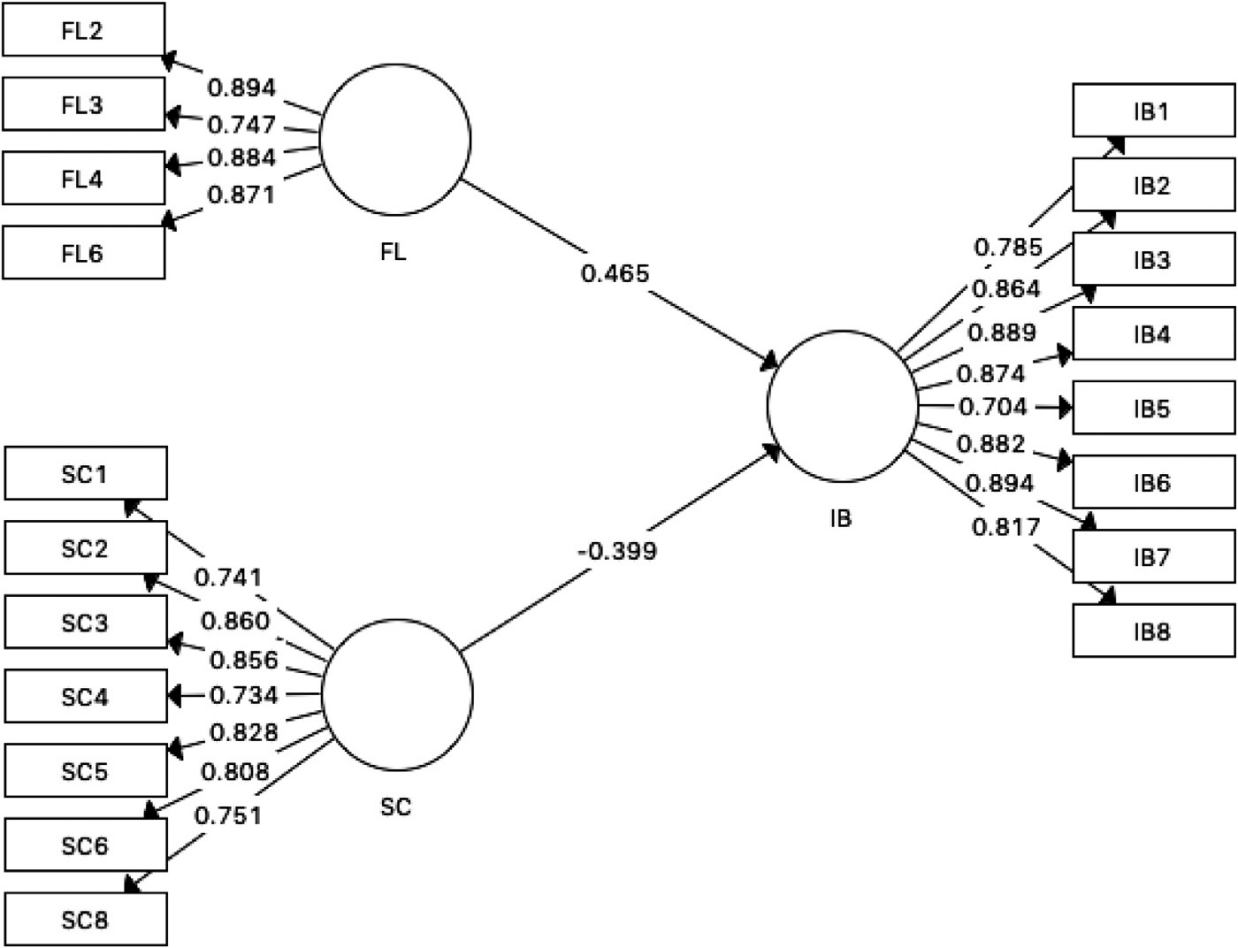
Final Model.

The final model was developed after the second analysis (Fig. 2). The model comprised of three variables (i.e. FL, SC and IB) with 19 items. PLS-SEM analysis using SmartPLS v.3.3.2 program was applied to evaluate and estimate the model. The outer loading for each item is presented in Fig. 2.

**Table 4:** Result of the second PLS-SEM algorithm analysis.

Variable	Cronbach's Alpha	CR	AVE
Financial literacy	0.872	0.913	0.724
Self-control	0.905	0.925	0.638
Investment behaviour	0.94	0.951	0.707

## Ethical statement

Informed consent was obtained from the participants prior to the survey.

**Table:** 

Investment behaviour Instrument					
**Construct**					
***Financial Literacy***	**Strongly agree**	**Agree**	**Neutral**	**Disagree**	**Strongly disagree**
I have better understanding of how to invest my money.					
I have better understanding of how to manage my credits use.					
I have the ability to maintain financial records for my income and expenditure.					
I can manage my money easily					
I have better understanding of financial instruments (e.g. Bonds, stock, T-bill, time value of money, future contract, option and etc.)					
I have the ability to prepare my own budget weekly and monthly					
***Self-Control***					
I don't save, because I think it is too hard.					
I enjoy spending money on things that aren't practical.					
When I get money, I always spend it immediately (within 1 or 2 days).					
‘I see it, I like it, I buy it’ describe me.					
‘Just do it’ describes the way I buy things.					
‘Buy now, think about it later’ describe me.					
I always failed to control myself from spending money.					
I am more concerned with what happens to me in short run than in long run.					
When I set having goals for myself, I rarely achieve them.					
***Peer Influence***					
As far I know, some of my friends regularly do save with a saving account.					
I always discuss financial management issue (saving) with my friends.					
I always discuss financial management issue (investment) with my friends.					
I always spend my leisure time with my friends.					
I always involve in money spending activities with friends.					
I always follow the information about investment growth.					
***Investment behaviour***					
I put money aside on a regular basis for the future.					
In order to invest, I often compare prices before I make purchase.					
In order to invest, I often consider whether the stock prices are valuable when I sell it.					
In order to invest, I often understanding the fundamental analysis.					
I always have money available in the event of my failed investment.					
In order to invest, I plan to manage my expenses.					
I save my money in order to do investment.					
I invest to achieve certain goals.					
I have some investment account in money market and also capital market.					

## Declaration of Competing Interest

The authors declare that they have no known competing financial interests or personal relationships which have, or could be perceived to have, influenced the work reported in this article.

## Appendix Supplementary materials

Image, application 1
